# Lower obstetrician and gynecologist (OBGYN) supply in abortion-ban states, despite minimal state-level changes in the 2 years post-*Dobbs*

**DOI:** 10.1093/haschl/qxae162

**Published:** 2024-11-27

**Authors:** Julia Strasser, Ellen Schenk, Qian Luo, Candice Chen

**Affiliations:** Fitzhugh Mullan Institute for Health Workforce Equity, Department of Health Policy and Management, Milken Institute School of Public Health, George Washington University, Washington, DC 20037, United States; Fitzhugh Mullan Institute for Health Workforce Equity, Department of Health Policy and Management, Milken Institute School of Public Health, George Washington University, Washington, DC 20037, United States; Fitzhugh Mullan Institute for Health Workforce Equity, Department of Health Policy and Management, Milken Institute School of Public Health, George Washington University, Washington, DC 20037, United States; Fitzhugh Mullan Institute for Health Workforce Equity, Department of Health Policy and Management, Milken Institute School of Public Health, George Washington University, Washington, DC 20037, United States

**Keywords:** Dobbs, workforce, OBGYNs

## Abstract

Since the *Dobbs v Jackson Women's Health Organization* Supreme Court decision in June 2022, emerging reports suggest that the obstetrician and gynecologist (OBGYN) workforce is moving into states without abortion bans. Using a large national administrative database, we identified OBGYNs enrolling in new states from July 2017 through June 2024. We used difference-in-differences (DID) analyses to estimate the effect of *Dobbs* on enrollments by state abortion policy (ban vs no ban). Enrollments in ban states were lower than in no-ban states in most academic years (June–July) throughout the study period. In the 2 years post-*Dobbs*, DID models found no significant differences in enrollments in ban states relative to no-ban states. These findings indicate minimal state-level shifts in the OBGYN workforce following *Dobbs*. Past research has found that a complex constellation of factors drives physician movement, including state licensure, job availability, income, spousal job opportunities, and social support. While the effect of the *Dobbs* decision on the workforce are likely to be significant, the full impacts on the workforce will take years to fully unfold.

## Introduction

The *Dobbs v Jackson Women's Health Organization* decision is changing the national landscape of reproductive health care, affecting both individuals seeking care and the clinicians providing it. A growing number of state-level restrictions on abortion now include punitive policies for the clinical workforce, such as jail time, civil prosecution,^1,2^ and loss of medical licensure.^3^ These restrictions are layered onto growing threats to abortion providers’ physical safety, including death threats, stalking, assault and battery, and clinic invasions,^4^ and to worsening moral distress and injury.^3,5,6^ National news has raised concerns over an “exodus” of clinicians, particularly obstetrician and gynecologist (OBGYN) physicians, as a result of these state policies and threats.^7-9^

Early evidence suggests that clinicians are already considering these changing state policies in making career and job decisions. In the 2023 national residency match, the overall number of US MD applicants to OBGYN residency programs dropped by 5.2% from the previous year, with the greatest decreases in states with complete abortion bans (10.5%).^10^ In a survey of OBGYN residents graduating in 2023, residents who had planned to practice in abortion-restrictive states were 8.5 times more likely to change their intended state of practice post-*Dobbs* compared with those planning to practice in nonrestrictive states. In open-ended questions, some residents reported that the developing abortion restrictions meant they chose not to move back to their home states as planned.^11^ In a November 2022 survey of OBGYNs, 63% of respondents said they would be unlikely to consider a position in an abortion-restrictive state.^12^ Over time, the inability of abortion-restrictive states to recruit new and existing clinicians will exacerbate widening health workforce disparities, with negative consequences for health care access, quality, and outcomes.

The workforce providing abortion care includes multiple types of clinicians—OBGYNs, family medicine physicians, advanced practice nurses, and others.^13^ While primary care providers and advanced practice clinicians undoubtedly are experiencing fallout from the *Dobbs* decision as well, OBGYNs currently make up the largest proportion of abortion provider types,^13^ and a loss of the OBGYN workforce due to abortion restrictions has potential spillover effects into the full scope of their services. As many predicted before the *Dobbs* decision,^14^ and as clinicians continue to report since the *Dobbs* decision,^5,15^ restrictions on abortion have immediate implications for other pregnancy-related care, such as treating ectopic pregnancies and miscarriages, and US regions with lower per-population availability of maternal health providers already have higher maternal mortality rates than the national average.^16^ Losing OBGYNs from a state not only means losing abortion providers but also losing physicians who can provide maternal health care, contraception, and treatment of complex obstetric and gynecological conditions.^17^

Potential shifts in the workforce must also be considered in the context of the existing maldistribution of providers. While 16% of the US population live in rural areas, only 8% of primary care providers (including OBGYNs) practice in these areas.^18^ The growing trend of hospital closures or obstetric unit closures, especially in rural areas, is an important contributor to geographic distribution. Between 2004 and 2014, 179 rural counties lost hospital-based obstetric services, leaving more than half of rural US counties with no hospital-based obstetric services.^19,20^ These hospital closures could drive clinician movement out of state. Some states have already seen a drop in the number of OBGYNs who accept Medicaid, which covers nearly half of births in the United States.^21^ Iowa, for example, lost approximately 10% of their Medicaid OBGYN workforce from 2016 to 2019.^22^ Notably, states with greater abortion restrictions are also largely rural.^23,24^ If OBGYNs leave states that already have fewer providers due to factors that predate *Dobbs*, workforce shortages in these states will become further exacerbated.

Despite rising concerns over an “exodus” of OBGYN physicians from abortion-restrictive states, more research is needed to describe whether there are new and substantial geographic shifts in the OBGYN workforce post-*Dobbs*. This study uses a large national administrative provider database to provide an early examination of geographic movement of OBGYNs up to 2 years post-*Dobbs*.

## Data and methods

### Data and outcomes

We analyzed the Medicare Provider Enrollment, Chain, and Ownership System (PECOS), an administrative dataset of over 2 million clinicians. PECOS enrollment is required for clinicians to bill Medicare, incentivizing timely enrollment even for low- or no-volume Medicare providers (such as OBGYNs and pediatricians) who work in organizations that otherwise bill Medicare. The PECOS dataset includes 48 892 OBGYNs who were enrolled during at least 1 quarter (Q) between July 1, 2017, and June 14, 2024. As of Q2 2024, there were 41 249 OBGYNs in PECOS, representing 96.2% of the OBGYNs listed in the American Medical Association (AMA) Masterfile for the same period, excluding OBGYN residents, administrative personnel, and researchers.^25^

**Figure 1.**
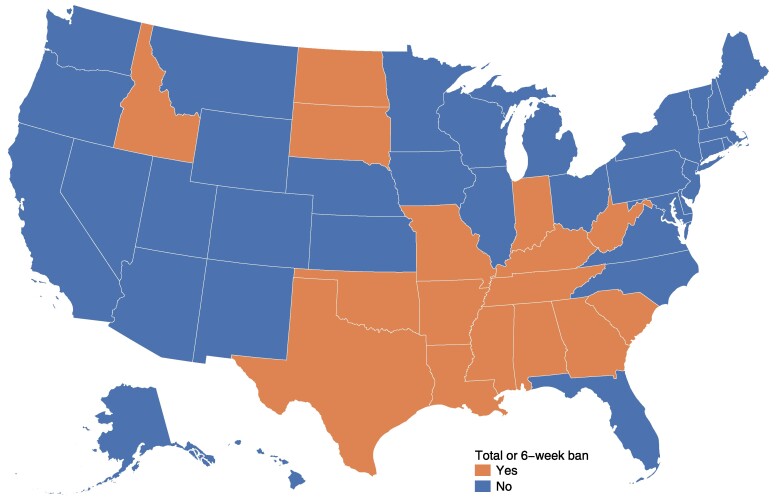
Abortion policies in the United States. Source: Authors’ analysis of Guttmacher Institute's State Bans on Abortion Through Pregnancy (see note 27 in text). States are classified as having a total or 6-week ban if the ban had been in place for at least 6 months as of July 1, 2024. Ban states include Alabama, Arizona, Georgia, Idaho, Indiana, Kentucky, Louisiana, Missouri, Mississippi, North Dakota, Oklahoma, South Carolina, South Dakota, Tennessee, Texas, and West Virgina.

When clinicians move across state lines or start practicing in a new state, there is a new PECOS enrollment record in the new state, allowing for analysis of clinician movement in nearly real time.^26^ We classified OBGYNs into 2 enrollment type categories: initial and existing. Initial enrollments reflect newly entering clinicians post-residency. Existing enrollments reflect clinicians who were previously enrolled in PECOS, where the new enrollment represents either a full move or initiation of services in a new state. We then assessed movement in these 2 groups by state abortion policy. States were categorized as “ban states” if they had either a full or 6-week ban in effect for 6 months or more as of July 1, 2024, and as “no-ban” states if not (Figure 1)^27^; while there are a number of ways to categorize state-level abortion restrictions, and the presence or absence of a ban is not the only type of restriction, we hypothesized that bans are the most likely driver of movement out of a state, rather than other types (eg, restrictions on telehealth).

This study was approved by the George Washington University Institutional Review Board, which considered this study exempt from the need for informed consent as this is a study of secondary data.

### Statistical analysis

We first calculated monthly enrollments in our 2 categories of interest. As monthly enrollments are variable and exhibit seasonal trends, we conducted descriptive analyses of enrollments per 100 000 reproductive–aged female population (ages 15–44 years) by academic year (AY). We chose AY (July–June) because of the timing of the *Dobbs* decision in late June and because physicians generally complete their residency in June and enter the workforce in July or August. We report enrollments for AY 2017–2018 through AY 2023–2024 by enrollment type and state policy (ban vs no ban).

To estimate the impact of *Dobbs* on OBGYN movement in ban states vs no-ban states, we used a difference-in-differences (DID) approach. Our outcome variables were monthly initial enrollments per 1 000 000 population of females aged 15–44 years and monthly existing enrollments per 1 000 000 population of females aged 15–44 years. Population data were from 2021 American Community Survey 5-year estimates of reproductive health–aged females. The treatment variable was enrollment in a ban state following the *Dobbs* decision, a binary variable that equaled 1 in ban states after the *Dobbs* decision (June 2022–June 2024) and 0 otherwise.

We conducted event study (leads and lags) analyses of initial and existing enrollments of OBGYNs per population from 2017–2024 to examine plausibility of the parallel trends assumption of DID—that is, that trends in the control group (enrollments in no-ban states) and treated group (enrollment in ban states) were parallel in the pre-period. All regressions included month-year and state fixed effects with clustered standard errors at the state level. Significance testing was based on 2-sided tests with a threshold of .05 and performed in Stata, version 18.0 (StataCorp, College Station, TX, USA).

### Sensitivity analysis

We also conducted several different sensitivity analyses for the main DID regression model with alternative specifications: using quarterly data, rather than monthly; using an alternative classification of states’ abortion policies (states were classified as ban if they had existing trigger laws and/or pre-*Roe* abortion bans [18 states])^28^; and using May 2022, the month of the *Dobbs* decision leak, as the post-period indicator. Finally, we identified general surgeons as a control group and conducted triple-difference analyses. This specialty was used as a comparison group because their scope of practice is less likely to be affected by the *Dobbs* decision, and the practice state is therefore likely to be independent of state policies on abortion. This analysis used a 3-way interaction term combining changes in initial and existing enrollments post-*Dobbs* (first difference), across states with and without abortion bans (second difference), and for OBGYNs relative to general surgeons (third difference).

## Results

### Descriptive analysis

**Figure 2:**
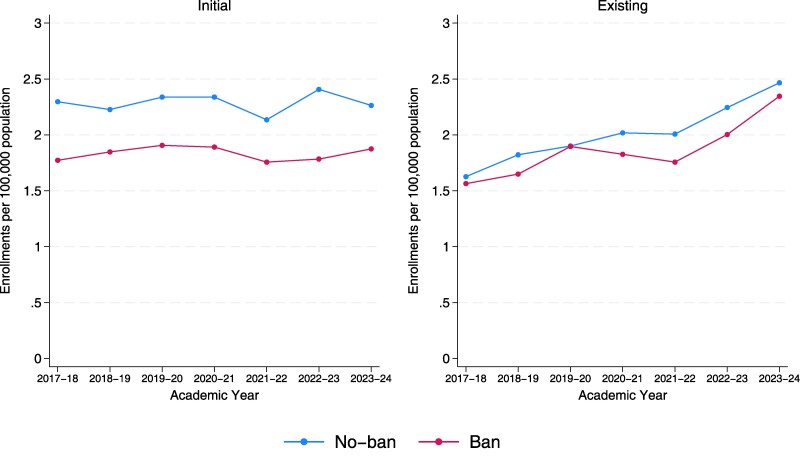
. Initial and existing enrollments among obstetrician and gynecologists (OBGYNs) by academic year and state abortion policy, 2017–2024. Source: Authors’ analysis of Medicare Provider Enrollment, Chain, and Ownership System. Per-population is enrollment count per 100 000 population of reproductive health–aged females (15–44 years) is 45 980 393 in no-ban states and 18 668 781 in ban states as of 2021.

Figure 2 shows per-population OBGYN enrollments by AY (July–June) for both enrollments of new physicians (initial) and new enrollments of practicing physicians (existing). From July 2017 through June 2024, there were 9756 initial and 8912 existing enrollments of OBGYNs, with 75.4% and 72.7% of enrollments occurring in no-ban states, respectively. Initial and existing enrollments per-population were higher in no-ban states compared with ban states for both initial and existing OBGYNs for all years except for 2019–2020. Initial enrollments show year-to-year variation in ban and no-ban states, ranging from 1.76 to 1.91 per 100 000 reproductive health–aged females in ban states and 2.14 to 2.41 per 100 000 reproductive health–aged females in no-ban states. Enrollments of existing OBGYNs per 100 000 reproductive health–aged females appeared to be increasing over the time period in both ban and no-ban states, from 1.56 in AY 2017–2018 to 2.35 in AY 2023–2024 in ban states and 1.63 in AY 2017–2018 to 2.47 in AY 2023–2024 in no-ban states. Annual enrollment counts for the full study period are available in Appendix Table A1 (to access the Appendix, click on the Details tab of the article online).

### DID analysis

In the 2-year post-*Dobbs* period compared with the pre-*Dobbs* period, the DID models found no significant effects for either initial (−0.26 enrollments per 1 000 000 population per state per month; 95% CI: −0.58 to 0.07; *P* = .12) or existing (−0.33 enrollments per 1 000 000 population per state per month; 95% CI: −0.96 to 0.30; *P* = .30) enrollments in ban states (Table 1).

**Table 1.** Difference-in-differences analysis of initial and existing enrollments of OBGYNs by state abortion policy, July 2017–June 2024.

	Initial enrollments per 1 000 000 female population aged 15-44 years	Existing enrollments per 1 000 000 female population aged 15–44 years
*Dobbs* decision	−0.26 (−0.58 to 0.07)	−0.33 (−0.96 to 0.30)
Constant	2.00* (1.97 to 2.03)	2.64* (2.58 to 2.70)
Observations	4284	4284

Source: Authors’ analysis of Medicare Provider Enrollment, Chain, and Ownership System. A total of 4284 observations are the number of state-months from 2017–2024. The constant represents the estimated average initial or existing enrollments of OBGYNs per 1 000 000 population of 1 of the no-ban states in the comparison group pre-*Dobbs* decision. 95% CIs were estimated using clustered SEs in parentheses. *P < .01.

Abbreviation: OBGYN, obstetrician and gynecologist.

Our event study analyses found that the parallel trend assumption held before the *Dobbs* decision, as there were no statistically significant differences in initial or existing enrollments between no-ban and ban states before June 2022 (Appendix Table A2).

### Sensitivity and robustness checks

Models with quarterly time periods, using the *Dobbs* leak as the post-period indicator and different state classifications, found the same trends as the main model—that is, no statistically significant differences (Appendix Tables A3–A5). Triple-difference estimates found that *Dobbs* was associated with no significant changes in either initial or existing enrollments of OBGYNs in ban states relative to general surgeons in no-ban states; however, there were significantly higher initial enrollments of OBGYNs per-population relative to general surgeons throughout the time period (Appendix Table A6).

## Discussion

Overall, we did not find significant changes in enrollments for initially enrolling or existing OBGYNs over a 2-year period post-*Dobbs*. For both initial and existing OBGYNs, there were higher per-population enrollments of OBGYNs into no-ban states compared with ban states throughout the pre-*Dobbs* period. Triple-differences models found no excessive movement of OBGYNs relative to general surgeons for initial and existing enrollments in ban states compared with no-ban states.

Although we did not find significant differences between graduates entering the workforce for the first time compared with practicing clinicians in the existing workforce, we did find an increasing number of existing OBGYNs enrolling in new states, regardless of ban status, during our study period of 2017–2024, while the number of new entrants to the workforce was relatively flat. As the number of residency graduates is limited by the number of residency slots, the existing workforce is larger, and we would therefore expect greater absolute numbers of state-level movements. We also note that decision-making may be different for these 2 groups. Residency graduates entering the workforce are beginning their post-residency practice and are at a natural inflection point for choosing where to practice and live as they complete their training. As previously noted, national match data suggest that US medical graduates’ interest in OBGYN as a specialty and in programs in ban states decreased after the *Dobbs* decision,^10^ signaling potential shifts in the workforce as clinicians complete training and choose where and what to practice. It is also important to note, however, that, despite decreased interest, residency spots continue to be filled in these states as of the 2024 match. Historically, physicians have tended to practice in the state where they are trained^29^; however, if current residents preferentially choose states without abortion bans, maldistribution will worsen over time. In addition, qualitative research among residency graduates finds that concerns about their own or their partner's pregnancy plans play a role in choosing where to practice in the context of abortion restrictions.^11^ At the same time, emerging research suggests that some trainees are choosing to stay in restrictive states out of a commitment to their community but nonetheless express concerns about the impact of abortion restrictions on their medical education.^30^

Providers in the existing workforce face a different set of considerations that may include current family or partner obligations (eg, children in school, partner's job) that make relocating more difficult and time-consuming. Past research has found that a complex constellation of factors drives physician movement, including state licensure, job availability, income, spousal job opportunities, and social support.^31,32^ In addition, clinicians who wish to move to a new state may face delays due to the availability of jobs in their desired location and the required time to apply for a new job, obtain a license in a new state, and be credentialed in a new organization. The AMA recommends that physicians allow at least 2 months for state licensure and the Medical Group Management Association reports up to 100 days for credentialing of new providers, suggesting the full process of starting a new job as a physician can take 6–12 months.^33,34^ This delay suggests that it will be critical to continue to track clinician movement over time, and account for changing policies, such as Interstate Medical Licensure Compacts, which can expedite physician licensing in other states and may make it easier for practicing physicians to move in the future. In addition, non-OBGYN physicians whose clinical practice is directly impacted by abortion restrictions (eg, emergency medicine physicians who increasingly treat conditions like management of miscarriage or ectopic pregnancy in emergency settings) may also be more likely to move as the full effects of the *Dobbs* decision continue to unfold. Additionally, in a state or community with a low number of OBGYNs pre-*Dobbs*, the loss of even a single provider may have profound effects on access to care for that community.

State-level physician workforce is ultimately driven by who enters and who exits, and this study focused only on entrance. Furthermore, movement is a single indicator of pressures on the workforce but is by no means the only measure of it. Across the clinical workforce, there is growing recognition of burnout and moral injury. For abortion providers, including OBGYNs, there is documented evidence on the moral distress and moral injury that these clinicians feel in abortion-restrictive environments.^3,5,6^ Increases in moral injury and burnout may lead to greater turnover, or “exodus,” in the future—but we must also consider them as important outcomes in their own right, and future research should prioritize these as key measures of the health of the workforce.

### Limitations

There are limitations to this analysis. An important limitation of PECOS is that organizations and individuals have little incentive to disenroll and an enrollment in a new state does not necessarily mean exit from the prior state. Therefore, we focus on movement into states and cannot identify either complete exit from practice or from a state. In addition, physicians may be enrolling in a practice site in a new state without moving to that state (eg, as Locums clinicians), and we are not able to differentiate those types of movements here. Workforce patterns also take years to fully develop, and these data only describe movement through 2 years post-*Dobbs*. Future data will be needed to tell the full story of both entry and exit in 2023 and later. We are also likely capturing clinicians who are shifting their services to a new state while still practicing in their previous state (eg, providing contraception through telehealth to patients in a different state).

In addition, policy changes outside of abortion restrictions may play a role unrelated to abortion; for example, many of the states restricting abortion are also restricting gender-affirming care and/or passing anti-diversity legislation.^35^ In 2022–2023, the workforce continued to grapple with lingering effects of the COVID-19 pandemic on the workforce, with evidence of increasing physician turnover post–COVID-19.^36^ States that banned abortion had lower COVID-19 vaccination rates and excess mortality, which may contribute to movement across the entire clinical workforce.^37^

## Conclusion

This research provides an early look at national-level OBGYN movement into new states, by state abortion policy, through the first 2 years post-*Dobbs*. The ramifications of *Dobbs* for the clinical workforce are monumental and still unfolding. Past research has shown that the OBGYN workforce is critical for maternity outcomes, and that maternity outcomes are worsening in recent years—particularly for people of color. If OBGYN movement increases in future years, with higher concentrations in states with fewer abortion restrictions, existing disparities in both obstetrical health and gynecological health will become further magnified. Future research should continue to examine workforce movement, as well as the effects on quality of care and moral injury among clinicians.

## Acknowledgments

The authors wish to thank Jenny O'Donnell, ScD for her helpful input on this study.

## Supplementary Material

qxae162_Supplementary_Data
