# Supplementary material for: Lower obstetrician and gynecologist (OBGYN) supply in abortion-ban states, despite minimal state-level changes in the 2 years post-Dobbs
Source: Health Aff Sch. 2024 Nov 27;2(12):qxae162. doi: 10.1093/haschl/qxae162 (PMC11638721; doi:10.1093/haschl/qxae162)
Supplement: qxae162_Supplementary_Data [file qxae162_supplementary_data.zip › Appendix (1).docx]

**Appendix**

**Appendix Table A1.** Initial and Existing Enrollments Among OBGYNs by Academic Year and State Abortion Policy, 2017 – 2024.

**Appendix Table A2**. Leads and Lags Analysis of *Dobbs* Decision on Initial and Existing Enrollments of OBGYNs in Abortion Ban States versus No-Ban States.

**Appendix Table A3.** Sensitivity Analyses of Difference-in-Differences Analysis of Initial and Existing Enrollments of OBGYNs by State Abortion Policy, Q3 2017 – Q2 2024.

**Appendix Table A4.** Sensitivity Analyses of Difference-in-Differences Analysis of Initial and Existing Enrollments of OBGYNs by State Abortion Policy, July 2017- June 2024.

**Appendix Table A5.** Sensitivity Analyses of Difference-in-Differences Analysis of Initial and Existing Enrollments of OBGYNs by Alternative Specification for Abortion Policy, July 2017- June 2024.

**Appendix Table A6.** Triple-Difference Analyses of Initial and Existing Enrollments of OBGYNs Compared to General Surgeons by State Abortion Policy, July 2017- June 2024.

**Appendix Table A1.** Initial and Existing Enrollments Among OBGYNs by Academic Year and State Abortion Policy, 2017 – 2024.

|  | **Initial** | | | | **Existing** | | | |
| --- | --- | --- | --- | --- | --- | --- | --- | --- |
| **Academic**  **Year** | **No-ban** | | **Ban** | | **No-ban** | | **Ban** | |
|  | **Count** | **Per pop** | **Count** | **Per pop** | **Count** | **Per pop** | **Count** | **Per pop** |
| 2017-18 | 1,056 | 2.30 | 331 | 1.77 | 748 | 1.63 | 292 | 1.56 |
| 2018-19 | 1,024 | 2.23 | 345 | 1.85 | 838 | 1.82 | 308 | 1.65 |
| 2019-20 | 1,075 | 2.34 | 356 | 1.91 | 874 | 1.90 | 354 | 1.90 |
| 2020-21 | 1,075 | 2.34 | 353 | 1.89 | 928 | 2.02 | 341 | 1.83 |
| 2021-22 | 982 | 2.14 | 328 | 1.76 | 923 | 2.01 | 328 | 1.76 |
| 2022-23 | 1,107 | 2.41 | 333 | 1.78 | 1,032 | 2.24 | 374 | 2.00 |
| 2023-24 | 1,041 | 2.26 | 350 | 1.87 | 1,134 | 2.47 | 438 | 2.35 |

Source: Authors’ analysis of Medicare Provider Enrollment, Chain, and Ownership System

Note: Per population is enrollment count per 100,000 population of reproductive health aged females (15-44) is 45,980,393 in no-ban states and 18,668,781 in ban states as of 2021.

**Appendix Table A2.** Leads and Lags Analysis of *Dobbs* Decision on Initial and Existing Enrollments of OBGYNs in Abortion Ban States versus No-Ban States.

| **Leads** | **Initial Enrollments per 1,000,000 Female Pop Aged 15-44** | **Existing Enrollments per 1,000,000 Female Pop Aged 15-44** |
| --- | --- | --- |
| July 2017 | -0.24 (-1.52 to 1.05) | 0.37 (-1.35 to 2.09) |
| August 2017 | 0.41 (-3.48 to 4.30) | 1.45 (-0.65 to 3.55) |
| September 2017 | -1.71 (-3.94 to 0.52) | 0.01 (-1.85 to 1.86) |
| October 2017 | -0.55 (-2.28 to 1.18) | 0.79 (-0.52 to 2.10) |
| November 2017 | -0.17 (-1.26 to 0.92) | 1.57 (-0.86 to 4.01) |
| December 2017 | -0.15 (-1.46 to 1.16) | 0.09 (-1.59 to 1.77) |
| January 2018 | -0.16 (-1.04 to 0.71) | -0.08 (-2.87 to 2.72) |
| February 2018 | 0.14 (-0.56 to 0.84) | 0.87 (-1.14 to 2.88) |
| March 2018 | -0.31 (-1.21 to 0.59) | 1.49 (-0.49 to 3.47) |
| April 2018 | 0.25 (-0.56 to 1.05) | 0.29 (-1.86 to 2.43) |
| May 2018 | 0.10 (-0.80 to 0.99) | 0.65 (-1.04 to 2.33) |
| June 2018 | -0.18 (-2.03 to 1.67) | 0.48 (-1.61 to 2.58) |
| July 2018 | -0.94 (-2.61 to 0.72) | 1.27 (-0.99 to 3.53) |
| August 2018 | -1.41 (-3.72 to 0.90) | 1.50 (-0.91 to 3.92) |
| September 2018 | -1.36 (-3.24 to 0.53) | 1.31 (-1.30 to 3.92) |
| October 2018 | -1.31 (-3.77 to 1.16) | 0.44 (-1.73 to 2.62) |
| November 2018 | -0.02 (-2.59 to 2.54) | -0.18 (-1.78 to 1.43) |
| December 2018 | -0.04 (-0.75 to 0.68) | 1.43 (-0.74 to 3.60) |
| January 2019 | 0.06 (-1.11 to 1.23) | -0.77 (-3.38 to 1.84) |
| February 2019 | 0.21 (-0.87 to 1.28) | 0.13 (-1.86 to 2.12) |
| March 2019 | 0.23 (-0.64 to 1.09) | 0.81 (-1.56 to 3.18) |
| April 2019 | -0.02 (-0.85 to 0.81) | 0.03 (-1.97 to 2.04) |
| May 2019 | -0.14 (-1.64 to 1.35) | -0.76 (-2.61 to 1.10) |
| June 2019 | 0.60 (-1.29 to 2.49) | -0.44 (-2.20 to 1.32) |
| July 2019 | -1.72 (-3.76 to 0.32) | 0.62 (-1.32 to 2.55) |
| August 2019 | -1.82 (-4.30 to 0.67) | 0.82 (-1.53 to 3.17) |
| September 2019 | -1.04 (-3.18 to 1.11) | 1.14 (-1.20 to 3.48) |
| October 2019 | -1.74 (-4.40 to 0.91) | 0.48 (-1.50 to 2.46) |
| November 2019 | -0.74 (-1.73 to 0.25) | 0.15 (-1.56 to 1.87) |
| December 2019 | 0.26 (-0.96 to 1.48) | 2.13 (-0.32 to 4.57) |
| January 2020 | 0.82 (-1.08 to 2.73) | -1.69 (-4.40 to 1.01) |
| February 2020 | -0.33 (-1.06 to 0.39) | 1.95 (-0.70 to 4.60) |
| March 2020 | 0.48 (-0.58 to 1.54) | 0.04 (-2.56 to 2.64) |
| April 2020 | -0.61 (-1.65 to 0.43) | 0.92 (-1.45 to 3.28) |
| May 2020 | 0.05 (-0.93 to 1.02) | 0.48 (-1.53 to 2.49) |
| June 2020 | 1.25 (-0.17 to 2.66) | -0.49 (-2.41 to 1.42) |
| July 2020 | -0.67 (-3.06 to 1.71) | 1.69 (-0.84 to 4.22) |
| August 2020 | -2.15** (-4.17 to -0.12) | 0.72 (-0.75 to 2.20) |
| September 2020 | -1.46 (-4.61 to 1.70) | -0.65 (-2.42 to 1.12) |
| October 2020 | -1.87 (-4.48 to 0.73) | 0.52 (-1.34 to 2.39) |
| November 2020 | 0.07 (-0.95 to 1.09) | 0.80 (-1.38 to 2.98) |
| December 2020 | -0.12 (-1.11 to 0.87) | 0.77 (-1.13 to 2.66) |
| January 2021 | 1 [Reference] | 1 [Reference] |
| February 2021 | -0.14 (-0.97 to 0.69) | 0.52 (-1.39 to 2.44) |
| March 2021 | 0.35 (-0.41 to 1.10) | 0.25 (-2.13 to 2.63) |
| April 2021 | 0.26 (-0.48 to 1.01) | 1.18 (-0.55 to 2.91) |
| May 2021 | -0.15 (-1.13 to 0.82) | -0.38 (-1.90 to 1.15) |
| June 2021 | -0.66 (-2.60 to 1.29) | -0.93 (-2.89 to 1.03) |
| July 2021 | -1.42 (-3.09 to 0.24) | 0.78 (-1.25 to 2.80) |
| August 2021 | -2.14 (-4.79 to 0.51) | -0.42 (-2.65 to 1.81) |
| September 2021 | 0.57 (-2.23 to 3.37) | 0.06 (-2.59 to 2.70) |
| October 2021 | -0.57 (-1.61 to 0.47) | 0.12 (-1.68 to 1.92) |
| November 2021 | -0.45 (-1.52 to 0.61) | -0.54 (-2.92 to 1.85) |
| December 2021 | -0.23 (-1.16 to 0.70) | -0.01 (-1.72 to 1.71) |
| January 2022 | -0.83 (-2.18 to 0.52) | -0.22 (-2.84 to 2.40) |
| February 2022 | 0.20 (-0.47 to 0.86) | 0.01 (-1.65 to 1.67) |
| March 2022 | 0.69 (-0.36 to 1.74) | 1.48 (-0.66 to 3.63) |
| April 2022 | -0.06 (-0.79 to 0.67) | 0.23 (-2.19 to 2.64) |
| May 2022 | 0.52 (-0.48 to 1.53) | -0.20 (-1.78 to 1.39) |
| **Lags** |  |  |
| June 2022 | 0.05 (-1.17 to 1.28) | 0.72 (-1.44 to 2.87) |
| July 2022 | -1.02 (-2.54 to 0.49) | -1.08 (-3.47 to 1.31) |
| August 2022 | -1.64 (-3.96 to 0.68) | -2.18 (-4.48 to 0.13) |
| September 2022 | -3.25** (-6.46 to -0.04) | -2.00 (-4.63 to 0.63) |
| October 2022 | -0.20 (-1.45 to 1.05) | -1.50** (-2.90 to -0.10) |
| November 2022 | -0.88 (-3.02 to 1.25) | -0.24 (-2.96 to 2.47) |
| December 2022 | -0.39 (-1.48 to 0.71) | 1.38 (-1.04 to 3.80) |
| January 2023 | 0.01 (-0.71 to 0.74) | 0.10 (-1.76 to 1.96) |
| February 2023 | -0.51 (-1.48 to 0.47) | 0.73 (-0.80 to 2.26) |
| March 2023 | 0.16 (-0.78 to 1.11) | -0.16 (-2.58 to 2.27) |
| April 2023 | -0.16 (-1.07 to 0.74) | 1.03 (-0.98 to 3.04) |
| May 2023 | -0.52 (-1.60 to 0.56) | 1.12 (-0.78 to 3.02) |
| June 2023 | -0.75 (-2.08 to 0.58) | 0.78 (-1.46 to 3.01) |
| July 2023 | -0.30 (-1.90 to 1.29) | 0.62 (-1.36 to 2.59) |
| August 2023 | 0.21 (-2.08 to 2.50) | -1.16 (-2.91 to 0.59) |
| September 2023 | -2.36 (-4.89 to 0.17) | 0.38 (-1.20 to 1.97) |
| October 2023 | -3.30 (-8.20 to 1.61) | -1.19 (-3.35 to 0.97) |
| November 2023 | -1.30 (-3.39 to 0.79) | 2.57 (-2.24 to 7.38) |
| December 2023 | 0.68 (-1.27 to 2.63) | 0.28 (-1.87 to 2.43) |
| January 2024 | -0.15 (-1.07 to 0.77) | 0.77 (-1.17 to 2.72) |
| February 2024 | -0.38 (-1.16 to 0.40) | 0.24 (-1.55 to 2.04) |
| March 2024 | 0.08 (-0.64 to 0.79) | 0.55 (-2.34 to 3.44) |
| April 2024 | 0.26 (-0.51 to 1.03) | -0.42 (-2.81 to 1.98) |
| May 2024 | 0.01 (-0.90 to 0.91) | 0.60 (-1.31 to 2.50) |
| June 2024 | -0.18 (-1.03 to 0.67) | 0.51 (-2.18 to 3.21) |
| **Constant** | 2.08*** (1.08 to 3.08) | 2.01** (0.38 to 3.65) |
| **Observations** | 4,284 | 4,284 |

Source: Authors’ analysis of Medicare Provider Enrollment, Chain, and Ownership System

Note: 4,284 observations are the number of state-months from July 2017 – June 2024. 95% confidence intervals estimated using clustered standard errors in parentheses.

*** p<0.01, ** p<0.05

**Appendix Table A3.** Sensitivity Analyses of Difference-in-Differences Analysis of Initial and Existing Enrollments of OBGYNs by State Abortion Policy, Q3 2017 – Q2 2024.

|  | **Initial Enrollments per 1,000,000 Female Pop Aged 15-44** | **Existing Enrollments per 1,000,000 Female Pop Aged 15-44** |
| --- | --- | --- |
| ***Dobbs* decision** | -0.52 (-1.42 to 0.39) | -1.04 (-2.90 to 0.82) |
| **Constant** | 5.98*** (5.89 to 6.07) | 7.94*** (7.75 to 8.12) |
| Observations | 1,428 | 1,428 |

Source: Authors’ analysis of Medicare Provider Enrollment, Chain, and Ownership System

Note: 1,428 observations are the number of state-quarters from 2017 – 2024. The constant represents the estimated average initial or existing enrollments of OBGYNs per 1,000,000 population of one of the no-ban states in the comparison group pre-*Dobbs*. 95% confidence intervals estimated using clustered standard errors in parentheses.

*** p<0.01

**Appendix Table A4.** Sensitivity Analyses of Difference-in-Differences Analysis of Initial and Existing Enrollments of OBGYNs by State Abortion Policy, July 2017- June 2024.

|  | **Initial Enrollments per 1,000,000 Female Pop Aged 15-44** | **Existing Enrollments per 1,000,000 Female Pop Aged 15-44** |
| --- | --- | --- |
| ***Dobbs* decision leak** | -0.20 (-0.51 to 0.12) | -0.35 (-0.98 to 0.28) |
| **Constant** | 1.99*** (1.96 to 2.03) | 2.64*** (2.58 to 2.71) |
| Observations | 4,284 | 4,284 |

Source: Authors’ analysis of Medicare Provider Enrollment, Chain, and Ownership System

Note: 4,284 observations are the number of state-months from 2017 – 2024. The constant represents the estimated average initial or existing enrollments of OBGYNs per 1,000,000 population of one of the no-ban states in the comparison group pre-*Dobbs* decision leak. 95% confidence intervals estimated using clustered standard errors in parentheses.

*** p<0.01

**Appendix Table A5.** Sensitivity Analyses of Difference-in-Differences Analysis of Initial and Existing Enrollments of OBGYNs by Alternative Specification for Abortion Policy, July 2017- June 2024.

|  | **Initial Enrollments per 1,000,000 Female Pop Aged 15-44** | **Existing Enrollments per 1,000,000 Female Pop Aged 15-44** |
| --- | --- | --- |
| ***Dobbs* decision** | -0.15 (-0.48 to 0.17) | -0.39 (-1.03 to 0.25) |
| **Constant** | 2.00*** (1.96 to 2.03) | 2.65*** (2.28 to 2.72) |
| Observations | 4,284 | 4,284 |

Source: Authors’ analysis of Medicare Provider Enrollment, Chain, and Ownership System

Note: 4,284 observations are the number of state-months from 2017 – 2024. The constant represents the estimated average initial or existing enrollments of OBGYNs per 1,000,000 population of one of the no-ban states in the comparison group pre-*Dobbs* decision. 95% confidence intervals estimated using clustered standard errors in parentheses. States with trigger laws and pre-*Roe* bans are AL, AZ, AR, ID, KY, LA, MI, MS, MO, ND, OK, SD, TN, TX, UT, WV, WI, WY.

*** p<0.01

**Appendix Table A6.** Triple-Difference Analyses of Initial and Existing Enrollments of OBGYNs Compared to General Surgeons by State Abortion Policy, July 2017- June 2024.

|  | **Initial Enrollments per 1,000,0000 Female Pop Aged 15-44** | **Existing Enrollments per 1,000,0000 Female Pop Aged 15-44** |
| --- | --- | --- |
| **S** | 0.49** (0.03 to 0.94) | 0.25 (-0.05 to 0.55) |
| **A x S** | -0.39 (-0.87 to 0.10) | -0.27 (-0.72 to 0.19) |
| **A x D** | -0.10 (-0.39 to 0.19) | 0.35 (-0.70 to 1.41) |
| **S x D** | -0.03 (-0.31 to 0.25) | 0.50 (-0.05 to 1.05) |
| **A x S x D** | -0.16 (-0.57 to 0.26) | -0.68 (-1.81 to 0.45) |
| **Constant** | 1.64*** (1.50 to 1.79) | 2.33*** (2.20 to 2.47) |
| Observations | 8,568 | 8,568 |

Source: Authors’ analysis of Medicare Provider Enrollment, Chain, and Ownership System

Note: 8,568 is the number of state-months by specialty (OBGYN and general surgeon) from 2017 – 2024. The constant represents the estimated average initial or existing enrollments of general surgeons per 1,000,000 population of one of the no-ban states in the comparison group pre-*Dobbs* decision. 95% confidence intervals estimated using clustered standard errors in parentheses. S = specialty (reference group is general surgeons); A = abortion policy (reference group is no-ban states); D = post-*Dobbs* (reference group is pre-*Dobbs* [July 2017 – May 2022])*.*

*** p<0.01, ** p<0.05
